# Atranorin, a Secondary Metabolite of Lichens, Exhibited Anxiolytic/Antidepressant Activity in Wistar Rats

**DOI:** 10.3390/life12111850

**Published:** 2022-11-11

**Authors:** Nicol Urbanska, Patrik Simko, Andrea Leskanicova, Martina Karasova, Zuzana Jendzelovska, Rastislav Jendzelovsky, Dajana Rucova, Mariana Kolesarova, Michal Goga, Martin Backor, Terezia Kiskova

**Affiliations:** 1 Institute of Biology and Ecology, Faculty of Science, Pavol Jozef Šafárik University in Košice, 04154 Košice, Slovakia; 2 Small Animal Clinic, University of Veterinary Medicine and Pharmacy in Košice, 04180 Košice, Slovakia; 3 Department of Biochemistry and Biotechnology, Institute of Biotechnology, Faculty of Biotechnology and Food Sciences, Slovak University of Agriculture, 94976 Nitra, Slovakia

**Keywords:** atranorin, depression, anxiety, Wistar rats, hippocampus, neurogenesis, stress, reactive oxygen species

## Abstract

Atranorin (ATR) is one of lichens’ many known secondary metabolites. Most current studies have investigated the various effects of ATR in vitro and only sporadically in vivo. The latest data indicate that ATR may have anxiolytic/antidepressive effects. This study aimed to analyze the potential of ATR in a depression-like state in male Wistar rats. Pregnant females were stressed by restricting their mobility in the final week of pregnancy three times a day for 45 min each, for three following days. After birth, progeny aged 60 days was stressed repeatedly. The male progeny was divided into three groups as follows: CTR group as a healthy control (n = 10), DEP group as a progeny of restricted mothers (n = 10), and ATR group as a progeny of restricted mothers, treated daily for one month with ATR (n = 10; 10 mg/kg of body weight, p.o.). Our results show that ATR acts as an antioxidant and markedly changes animal behavior. Concomitantly, hippocampal neurogenesis increases in the hilus and subgranular zone, together with the number of NeuN mature neurons in the hilus and CA1 regions. Our results indicate a potential antidepressant/anxiolytic effect of ATR. However, further studies in this area are needed.

## 1. Introduction

Depression ranks fourth among the world’s diseases after respiratory diseases, prenatal disorders, and AIDS [1]. In 2020, the global COVID-19 pandemic caused a 27.6% increase in depression and 25.6% increase in anxiety [2]. Depression is accompanied by several side effects, such as increased anxiety, physical pain, suicidal tendencies, or loss of concentration and interest, and thus, has an impact not only on behavior and mood but also on a person’s overall health and is more economically important [3]. In addition, mental illness is a risk factor for other diseases, such as ischemic heart disease and cancer [4]. Current antidepressant treatments have insufficient efficacy or tolerance, many side effects, and frequent relapse [5]. Serious side effects include suicidal tendencies and cardiovascular and memory disorders [6,7]. However, a variety of herbs and natural substances have been reported to have fewer adverse effects than conventional medications [8].

Atranorin (ATR) was first isolated by Hesse in 1898 [9]. After usnic acid, ATR is the second most studied cortical secondary metabolite [10,11] and is found in various species of lichens, such as *Stereocaulon caespitosum* [12], *Parmelia sulcata, Evernia prunastri* [12,13], *Cladina kalbii* [14], *Lethariella canariensis* [15], and *Stereocaulon alpinum* [16]. ATR has demonstrated antimicrobial [17], antitumor [18], cytotoxic [19,20], immunomodulatory [21], antiviral [22], anti-inflammatory [23], antioxidant [15,24], antinociceptive [14,25], and antifungal activities [26]. Some of the current studies that examined the different effects of ATR have been performed only sporadically in vivo. The analgesic and anti-inflammatory activity of ATR in vitro was demonstrated by its strong effect on cyclooxygenases 1 and 2 at a concentration of 45 μM [27] and leukotriene B4, with an IC50 value of 6.0 ± 0.4 µM [23]. A study by Melo et al. reported the antinociceptive effect of ATR using a writhing test in mice at concentrations of 200 and 400 mg/kg. Depending on the dose, they inhibited acetic acid by 52.6% and 61.3%, respectively, and the anti-inflammatory effect occurred after 15–30 min of the formalin test at a concentration of 400 mg/kg of ATR [25]. Microsomal prostaglandin E2 synthase-1 inhibited ATR at a concentration of 10 μM [28]. Reddy et al. demonstrated a neurotrophic effect on Neuro2A neuroblast cells in vitro. After the application of ATR (5 µM), a significant increase in the expression of the neurotrophic factors BDNF and NGF was observed using RT-PCR (semi-quantitative) [29]. A study by our group focused on the effects of ATR on behavioral and neuronal changes after peroral administration in female and male Sprague-Dawley rats [30]. Based on our previous results, ATR may exert anxiolytic/antidepressant effects. However, such effects of ATR in vivo have not been described yet. Therefore, our study aimed to determine the potential anxiolytic/antidepressant effects of ATR (10 mg/kg body weight) in Wistar rats.

## 2. Materials and Methods

### 2.1. Extract of Lichen and Preparation of ATR

The lichen *Pseudoevernia furfuracea* (L.) Zopf. was collected from the branches of pines and spruces in Špania dolina (48°49′ N, 19°08′ E), central Slovakia, 730 m a.s.l. The collected material was handled as previously described [30]. ATR was isolated according to a protocol described by Elečko et al. [31].

### 2.2. Laboratory Animals and Experimental Design

Parental generation: two Wistar males and four Wistar females (Department of Toxicology and Laboratory Animal Breeding ÚEFT SAV, Dobrá Voda, Slovakia) were used. The animals were kept under standard vivarium conditions at room temperature (21–24 °C), with a relative humidity of 50–65%, and a 12:12 h light:dark regimen. The animals were provided with tap water and pelleted food (Velaz, Únetice, Prague, Czech Republic) ad libitum according to EU animal-fed legislation and guidance. Parental females have mated with parental males. For further experiments, male progeny was used. The animals were handled by the guidelines established by Law No. 377 and 436/2012 of Slovak Republic for the Care and Use of Laboratory Animals and approved by the State Veterinary and Food Administration of the Slovak Republic (Approval Number: Ro-2866/16-221, Ro-2219/19-221/3).

Transcardial lavage was used to kill laboratory animals at the end of the experiment under deep anesthesia (400 mg/kg of chloral hydrate, i.p.). Perfusion via the left ventricle started with washing of blood vessels with 0.9% saline followed by 4% fresh paraformaldehyde solution. The brains were removed, weighed, post-fixed for 24 h in the same fixative, and transferred to 30% sucrose solution for cryoprotection. Coronal sections of the brain (33 µm width) were cut on a cryostat.

### 2.3. Induction of Depression in Animals

Pregnant females were divided into two experimental groups: control and “depression-like”. Females from the “depression” group were stressed using restriction of mobility in the final week of pregnancy using the following schema: three times a day for 45 min each, for three following days [32]. After birth, the male progeny was kept with their mothers for 30 days. They formed three groups: the CTR group as a progeny of control mothers (n = 10), the DEP group as a progeny of restricted mothers (n = 10), and the ATR group as a progeny of restricted mothers, which were treated with ATR after birth (n = 10).

ATR was administered daily *per os* at a dose of 10 mg/kg of body weight. It was freshly dissolved in 10% ethanol and administered for one month, starting at the age of one month.

### 2.4. Behavioral Tests

After one month of ATR administration, the rats were tested using the forced swim test (FST), open field test (OFT), and elevated plus-maze test (EPM), as described previously [30,33,34].

#### 2.4.1. Forced Swim Test

Rats were placed in the maze and allowed to swim. The duration of time spent immobile was measured along with the time spent climbing and swimming. Each trial lasted for 5 min.

#### 2.4.2. Open Field Test

In the OFT, locomotor activity as total traveled distance, average speed of each animal, time spent on the periphery, and time spent in the center were measured. We also observed exploratory behaviors in terms of rearing, defecating, and comfort behaviors when washing. At the start of the test, the rats were placed in the center of the apparatus. Each trial lasted 6 min. The progress of the animals in OFT was recorded and evaluated using the computerized video-tracking system Smart Junior (Panlab, Barcelona, Spain).

#### 2.4.3. Elevated Plus-Maze Test

EPM was used to monitor anxiety-related behavior and partially detect the antidepressant effects of ATR. The level of anxiety as the time spent in the open arms of the labyrinth and the frequency of defecation, exploratory activity as the amount of rearing, comfort behavior as the number of washing acts, and locomotor activity as the number of passes through the center of the maze were recorded. Each trial lasted for 5 min.

### 2.5. Blood Collection

At the end of the experiment, blood samples were collected from *vena saphena* of each animal. Blood was collected in microtubes containing an appropriate volume of heparin. Blood plasma was prepared and stored at −80 °C for further analyses. Cortisol (ADVIA Centaur^®^ XP immunoassay system; Siemens for OrthoClinical Diagnostics, Inc., Singapore, Singapore) and adrenocorticotropic hormone (ACTH; Elecsys ACTH Test System, Cobas; BioLab Diagnostics, Naples, Italy) levels were measured.

### 2.6. Measurement of Reactive Oxygen Species in Leukocytes

Blood samples (100 μL) obtained from rats were drawn into heparin-treated tubes. The red blood cells were lysed using red blood cells lysis buffer (150 mM NH_4_Cl, 10 mM KHCO_3_, 0.1 mM EDTA, pH 7.4) for 3 min. The samples were then centrifuged at 200× *g* for 6 min. The pellet was then washed with 1 mM PBS-EDTA. Subsequently, each sample was divided into halves; one of the halves was stained and the other was used as an unstained autofluorescence control. Whole blood samples were stained with 10 μM 3 dihydrorhodamine-123 (DHR 123, Fluka, Buchs, Switzerland) for 20 min at room temperature in the dark as described previously [35]. The samples were measured using a BD FACSCalibur (BD) flow cytometer (Becton Dickinson, San Jose, CA, USA) with a 488 nm argon-ion excitation laser. Debris was eliminated by forward scatter and side scatter (FSC × SSC). Fluorescence was detected using 530/30 band-pass filter (FL-1) and quantified using the FlowJo software (Tree Star, Inc., Ashland, OR, USA). The level of reactive oxygen species (ROS) in leukocytes was expressed as a ratio of the DHR 123 fluorescence median to autofluorescence of unstained samples.

### 2.7. Immunohistochemical Staining of Proliferating Hippocampal Progenitors

According to Gerdes et al. and Kee et al., Ki67 is a reliable marker of the proliferation of normal and neoplastic cells [36,37]. Immunohistochemistry was performed on prepared coronal free-floating sections. Briefly, after incubation in blocking buffer, sections were incubated overnight at 4 °C with a primary antibody (Ki-67 (D3B5) rabbit mAb, #12202; dilution, 1:400; Cell Signaling Technology, Danvers, MA, USA). After washing, the sections were incubated with goat anti-rabbit IgG secondary antibody (Vector Laboratories, 1:200) for 1 h at room temperature. Sections were incubated in avidin-biotin-peroxidase complex (Vector Laboratories, Burlingame, CA, USA, Vestastain ABC kit) at room temperature for 1 h, and DAB (12 mM in PBS with 0.003% H_2_O_2_, Sigma-Aldrich, St Louis, MO, USA) was applied for 10 min. After dehydration, the sections were coverslipped using Permount (Fischer Scientific, Pittsburg, PA, USA).

### 2.8. Immunohistochemical Staining of Mature Neurons

Immunohistochemistry was performed on the prepared coronal free-floating sections to identify mature neurons. Briefly, the sections were incubated overnight at 4 °C with anti-NeuN antibody (MAB377, 1:500; Millipore, Bedford, MA, USA) in 0.1 M PBS (pH 7.4) with 0.3% Triton. After washing with 0.1 M PBS (pH 7.4) with 0.2% Triton, secondary anti-mouse IgG antibody (BA-2000, 1:200) was applied for 90 min at room temperature. After washing, ABC Elite (Vector Laboratories, Burlingame, CA, USA) was applied for 90 min, and then, the slides were rinsed with PBS and reacted with DAB (0.1 M Tris, 0.04% DAB, 0.033% H_2_O_2_); the reaction was stopped with phosphate buffer. The slides were dehydrated, cleared, and coverslipped for analysis [33].

The cells were counted using ImageJ software to evaluate the changes in neurogenesis and cell proliferation in the hippocampus.

### 2.9. Cell Counting

Photomicrographs of the brain slices were obtained using a microscope (Leica DM2500) and analyzed using the Image Tool (UTHSCSA, San Antonio, DX, USA) by a person blinded to the experimental conditions. The analysis was performed by quantifying the number of cells in every sixth cryostat section of the hippocampus. The number of NeuN-positive cells was counted within the different hippocampal areas: in the middle of the linear part of the CA1 region (at 200× magnification), in the whole area of the hilus (at 100× magnification), and in the granule cell layer (GCL) (at 200× magnification), as described previously [35]. The data for NeuN-positive cells in the linear structures, CA1 region, and GCL are expressed as the absolute number of cells for a chosen 400 μm part of each examined section. The number of Ki67-positive cells was counted in the whole SGZ and hilus of the *gyrus dentatus* (at 100× magnification).

### 2.10. Statistical Analysis

All statistical analyses were performed using GraphPad Prism 8.0.1 (GraphPad Software Inc., San Diego, CA, USA). All data were examined for normal distribution. One-way ANOVA test was used to see the statistical significance. Kruskal–Wallis test followed by Dunn’s multiple comparison test was performed. Data are presented as mean ± standard deviation (SD).

## 3. Results

### 3.1. Analyzing the Animal Behavior

**Figure 1:**
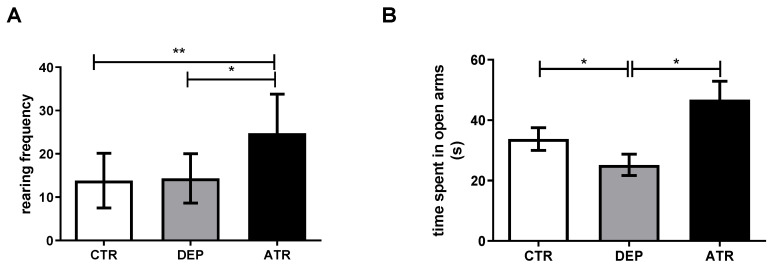
Rearing frequency (**A**) and the time spent in the open arms (**B**) in individual groups of control animals (CTR), depressive-like animals (DEP), and atranorin-treated animals (ATR) were evaluated using the elevated plus maze test. Values are presented as arithmetic mean ± SD. Significance is indicated by * *p* ˂ 0.05 and ** *p* ˂ 0.01.

**Figure 2:**
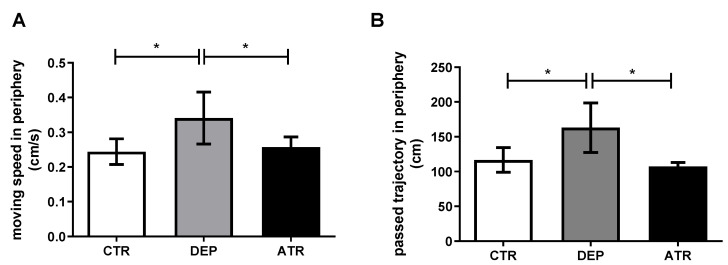
The moving speed (**A**) and trajectory passed in the periphery (**B**) in individual groups of control animals (CTR), depressive-like animals (DEP), and atranorin-treated animals (ATR) evaluated in the open-field test. Values are presented as arithmetic mean ± SD. Significance is indicated by * *p* ˂ 0.05.

During depression, a significantly decreased time spent in the open arms (*p* < 0.05) was observed in the EPM test (Figure 1), together with an increased mean speed (*p* < 0.05) and the trajectory passed in the periphery (*p* < 0.05) in the OFT (Figure 2A,B). However, after ATR treatment, there was a statistically significant increase in rearing frequency compared with the DEP group (*p* < 0.05) and CTR group (*p* < 0.01) and time spent in the open arms (*p* < 0.05) compared with DEP animals in the EPM (Figure 1). The moving speed (Figure 2A; *p* < 0.05) and passed trajectory (Figure 2B; *p* < 0.05) returned to the levels of CTR animals in the ATR group in the OFT. Healthy CTR animals behaved in a standard manner in the FST. However, depressed animals seemed to panic, and many of them began to drown. Therefore, testing was stopped.

### 3.2. Weights of Rat Brains

**Figure 3:**
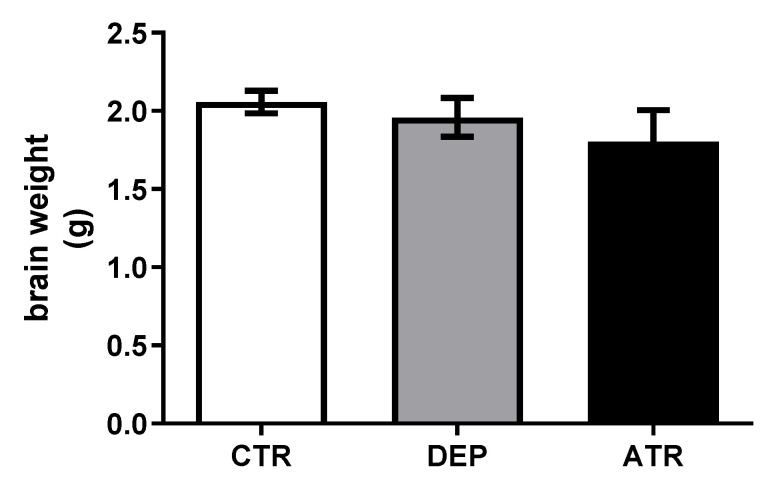
Brain weights (g) in individual groups of control animals (CTR), depressive-like animals (DEP), and atranorin-treated animals (ATR). Values are presented as arithmetic mean ± SD.

Brain weight was not influenced during depression (DEP) or ATR treatment (ATR) (Figure 3).

### 3.3. Analyzing the Level of Hippocampal Neurogenesis and Mature Neurons

#### 3.3.1. The Number of Ki67 Proliferative Cells

**Figure 4:**
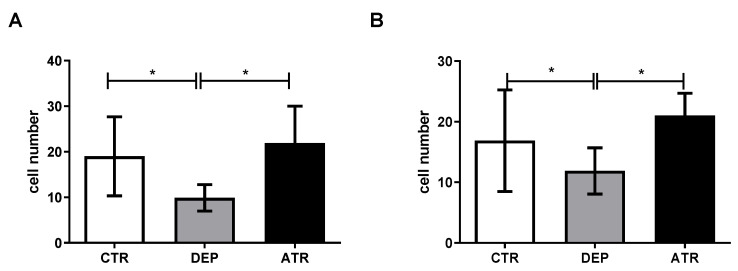
The number of proliferative Ki67-positive cells in the hilus (**A**) and subgranular zone (**B**) in individual groups of control animals (CTR), depressive-like animals (DEP), and atranorin-treated animals (ATR). Values are given as arithmetic mean ± SD. Significance is indicated by * *p* ˂ 0.05.

During depression, the cell number in the hilus and subgranular zone (SGZ) decreased significantly (Figure 4). However, ATR increased the number of proliferative Ki-67 cells in both the hippocampal zones (*p* < 0.05).

#### 3.3.2. The Number of NeuN Mature Neurons

**Figure 5:**
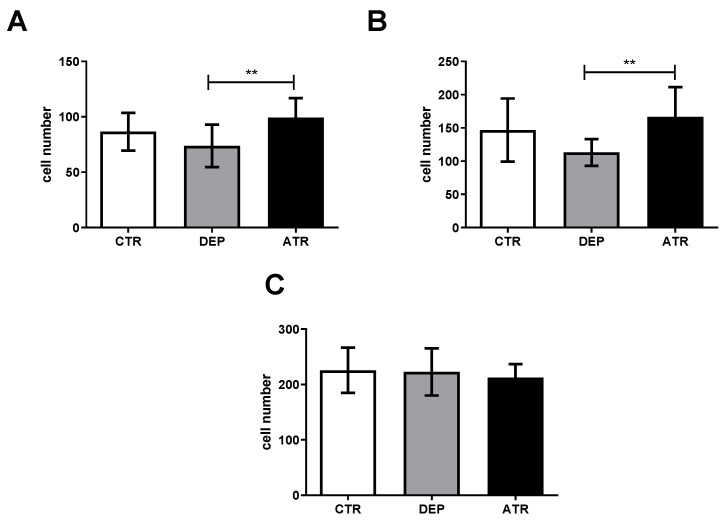
Number of NeuN-positive cells in the hilus (**A**), CA1 region (**B**), and granular cell layer (**C**) in individual groups of control animals (CTR), depressive-like animals (DEP), and atranorin-treated animals (ATR). Values are presented as arithmetic mean ± SD. Significance is indicated by ** *p* ˂ 0.01.

We did not observe any change in the number of NeuN-positive cells in the DEP group compared to the CTR group (Figure 5) in the hilus, GCL, and CA1 regions. In contrast, ATR treatment significantly increased the number of mature neurons in the hilus and CA1 region. The number of cells in the GCL did not change.

### 3.4. Stress Induced Changes in Hormones

**Figure 6:**
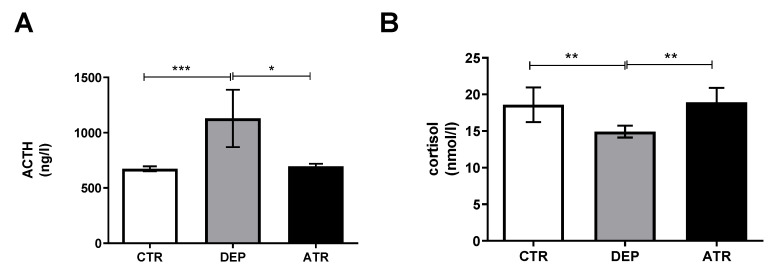
ACTH (**A**) and cortisol (**B**) levels in the blood of control animals (CTR), depressive-like animals (DEP), and atranorin-treated animals (ATR). Values are presented as arithmetic mean ± SD. Significance is indicated by * *p* ˂ 0.05, ** *p* ˂ 0.01, and *** *p* ˂ 0.001, respectively.

Glucocorticoids have been suggested to be involved in several stress-associated brain diseases [38,39]. Cortisol induces apoptosis in mature hippocampal neurons [40]. In response to stressful stimuli, corticotropin-releasing hormone is secreted into the hypothalamic-pituitary circulation, where it triggers ACTH release, which in turn stimulates the release of cortisol from the adrenal cortex [41]. Therefore, we analyzed the levels of cortisol and ACTH in the blood of experimental animals. As shown in Figure 6, the levels of ACTH (Figure 6A) and cortisol (Figure 6B) in the animals with depression (DEP group) changed significantly (*p* ˂ 0.001 and *p* ˂ 0.01, respectively). In contrast, ATR normalized the hormone levels to those in the CTR animals.

### 3.5. Reactive Oxygen Species in Leukocytes

**Figure 7:**
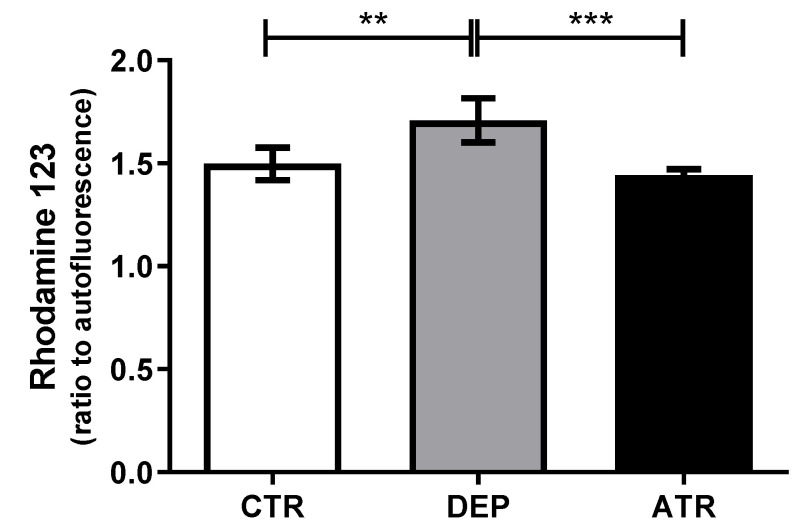
The level of reactive oxygen species in blood leukocytes in individual groups of control animals (CTR), depressive-like animals (DEP), and atranorin-treated animals (ATR). Values are presented as arithmetic mean ± SD. Significance is indicated by ** *p* ˂ 0.01 and *** *p* ˂ 0.01.

As shown in Figure 7, depression induced the release of free radicals (*p* < 0.01) compared with the CTR group. On the other hand, ATR was able to return the production of ROS to the level of CTR animals and drop the level in comparison with DEP males (*p* < 0.001).

## 4. Discussion

In recent years, there has been an increasing interest in the biological properties of ATR. Recent studies have investigated various effects of ATR predominantly in vitro and exceptionally in vivo. They focused mainly on the cytotoxic [42,43,44], antibacterial [26,45,46], and antioxidant [31,47,48] effects of ATR as well as its anti-inflammatory activities [27,49,50]. This study focused on whether ATR has the potential to treat depression.

After ATR application, rearing frequency and time spent in the open arms increased significantly compared to those in the DEP group. In contrast, rearing frequency increased markedly when compared to the CTR group. Based on a study by Simko et al., ATR administered to healthy animals at a dose of 10 mg/kg of body weight increased the time spent in the open arms in the EPM test when compared to both healthy male and female Sprague-Dawley rats. In addition, rearing frequency in the OFT increased in healthy animals treated with ATR [30]. The results of our study indicate that a decrease in anxiety occurs after the ATR application. In general, an increase in rearing frequency and time in the open arms indicates a decrease in anxiety in DEP animals [51,52]. Common depressive and anxiety states in animals and humans have been scientifically proven [53,54]. The reduction in exploratory behavior in animals in the EPM behavioral test indicates that ATR may act on their anxiety and depressive states. A decrease in exploratory behavior has also been observed in clinical studies of patients with depression [55]. During testing in the OFT, there was an increase in the moving speed and trajectory of depression-like animals in the periphery compared to the CTR group. This “chaotic” behavior could be described as anxious and may correspond to depressive-like state of DEP animals. The levels in the ATR group were comparable to those in the CTR group.

Recent experimental findings suggest that exploratory activity is dependent on the hippocampus and other areas of the medial temporal lobe. The hippocampus supports a set of memory processes that allow animals to intelligently and efficiently sample their environment and memory [56]. The link between neurogenesis and depression is best characterized by the neurogenic hypothesis, which considers a decrease in neurogenesis to be a result of depression; conversely, its restoration by antidepressants results in the recovery from the disease [57]. Exposure of monkeys and rats to prenatal stress impairs memory, increases the hypothalamic-pituitary axis, reduces hippocampal neurogenesis, and leads to depression and anxiety during the lifetime of mammals [58,59]. Under acute stress, neurogenesis levels off within 24 h, but after chronic stress, neurogenesis in the SGZ continues to decline [60]. The combination of repeated prenatal and postnatal stress was sufficient to induce anxiety- and depression-like states in this study. We evaluated the number of Ki67 proliferating cells in the hilus and SGZ, where the precursors of proliferating cells are located [61]. A decrease in Ki67 of proliferating cells occurred in both the hilus and SGZ in the DEP group compared with that in the CTR group. The decrease in proliferative activity during chronic stress could be due to an increase in the level of glucocorticoids, which correlated with the increase in cortisol in the blood of our experimental animals. This is in agreement with the findings of Czéh et al. [62]. Neural stem and progenitor cells are not characterized by glucocorticoids or mineralocorticoid receptors [63]. Glutamate can indirectly increase glucocorticoid levels, thereby reducing cell proliferation [64]. After ATR administration, the number of proliferating cells in both the SGZ and hilus in the ATR group increased significantly compared to that in the DEP group, together with the restored stress hormones levels, indicating the antidepressant effect of ATR.

It is known that proliferative hippocampal cells may differ into mature neurons [65]. Neuronal maturation may be accelerated by voluntary exercise and antidepressant treatment with fluoxetine [66]. In our experiment, we evaluated the presence and quantity of NeuN-positive cells (mature neurons), as described previously [67]. In the hilus and CA1 regions, we observed only a slight decrease in NeuN-positive cells compared with that in the CTR group. However, ATR significantly increased the number of mature neurons in the hilus and CA1 region. In addition, in an experiment on neuroblastoma cells (Neuro2A), physodic acid and ATR increased neurite length [68]. However, in the GCL, the number of NeuN-positive cells did not change in either the DEP or ATR group. This discrepancy may be caused by the calculation of positive cells only in a selected area of the GCL layer. Another explanation may be that the results may have been caused by the temporal maturity of the cells, as well as by the species of animals. For example, in mice, the maturation of GCL neurons takes place 2–3 weeks after the generation of neurons, whereby they acquire, though not completely, the electrophysiological properties of mature neurons, and only after 4–8 weeks do they connect to the hippocampal circuit. Dendrites extend into the *stratum moleculare*, where they receive inputs from the endothelial cortex [69,70,71]. In rats, complete maturation of the morphology occurs after four months [72].

During depression, the total brain weight may decrease as a consequence of a reduction in the volume of individual brain structures [73]. Volume reduction of some brain structures has been observed in patients with depression, such as in the amygdala, hippocampus, orbitofrontal cortex, and anterior cingulate cortex [74]. A similar study noted a volume reduction in frontal regions, such as the orbitofrontal cortex and anterior cingulate cortex, during depression. A smaller reduction has been observed in the prefrontal cortex [73]. However, other studies have reported increased amygdala volume in patients with depression [75,76]. In our study, the weight of the brain did not change in the DEP group compared with that in the CTR group. ATR itself did not change the brain weight of laboratory animals. This may have been caused by the length of the depression-like state of the animals.

Our results demonstrate the antioxidant properties of ATR. Another study found that the antioxidant potential of ATR was dependent on free radicals (hydroxyl radicals, superoxide radicals, hydrogen peroxide, and nitric oxide) at a concentration of 100 μg/mL in vitro [77]. A similar study by Siqueira et al. demonstrated the scavenging of free radicals by ATR at a concentration of 100 μg/mL [14]. ATR absorbed superoxide anion in vitro at an IC50 concentration of 169.65 µg/mL [78].

How ATR acts in the brain remains unclear. Currently, some secondary metabolites of the lichen *Hypogymnia physodes* from the *Parmeliaceae* family, such as ATR or physodic acid, may pass through the blood–brain barrier (BBB) [79]. For example, depsidone physodic acid and depsid evernic acid pass through the BBB either in the form of an extract or a pure substance. However, other secondary metabolites, such as depsidone salazinic acid, were not able to cross the BBB either in pure form or in the form of an extract, which may indicate a difference in the chemical structure of the compounds [28,80]. Alpha-glucans from lichens induce long-term potentiation (LTP) in the dentate gyrus of rats. LTP is mediated by peripheral activity. Alkyl chains in the chemical structure of lichen secondary metabolites can be a prerequisite for a better affinity to receptors and an increase in the lipophilic properties of molecules, which can allow better passage through the BBB [81,82]. (S)-5-Methylmellein was isolated from a lichenized endogenous fungus, which complied with Lipinski’s five rules in silico, thus demonstrating absorption through the gastrointestinal tract and passage through the BBB [83]. ATR fulfils Lipinski’s five rules, which means that it is highly absorbed by the gastrointestinal tract but probably does not pass through the BBB via the P-glycoprotein (Pgp) mechanism using docking studies [84]. However, the changes in the brains of rats found in this study, or in our previously published data [30], indicate a molecular mechanism by which ATR stimulates neurogenesis or induces behavioral changes.

The results of this study provide the first evidence of increased neurogenesis following ATR treatment in depression. Based on our observations, ATR is a potential lichen secondary metabolite for the treatment of depression and should be included in future studies.

## Data Availability

Not applicable.
